# Overexpression of TNFα induces senescence, autophagy and mitochondrial dysfunctions in melanoma cells

**DOI:** 10.1186/s12885-021-08237-1

**Published:** 2021-05-06

**Authors:** Silvia Tyciakova, Valeria Valova, Barbora Svitkova, Miroslava Matuskova

**Affiliations:** 1 Cancer Research Institute, Biomedical Research Center of Slovak Academy of Sciences, Dubravska cesta 9, 845 05 Bratislava, Slovakia; 2 Department of Genetics, Faculty of Natural Sciences, Comenius University, Mlynská dolina, Ilkovicova 6, 842 15 Bratislava, Slovakia

**Keywords:** TNFα, Melanoma, Senescence, Autophagy, Aldehyde dehydrogenase activity, Mitochondrial status, Cancer stem cell-related markers

## Abstract

**Background:**

Tumor necrosis factor alpha (TNFα) is a pleiotropic cytokine with both anti-tumorigenic and pro-tumorigenic activity, affecting tumor cell biology, the balance between cell survival and death. The final effect of TNFα is dependent on the type of malignant cells, with the potential to arrest cancer progression.

**Methods:**

In order to explain the diverse cellular response to TNFα, we engineered melanoma and colorectal carcinoma cell lines stably overexpressing this cytokine.

**Results:**

Under the *TNFα* overexpression, significant upregulation of two genes was observed: proinflammatory cytokine *IL6* gene in melanoma cells A375 and gene for pro-apoptotic ligand *TRAIL* in colorectal carcinoma cells HT29, both mediated by TNFα/TNFR1 signaling. Malignant melanoma line A375 displayed also increased autophagy on day 3, followed by premature senescence on day 6. Both processes seem to be interconnected, following earlier apoptosis induction and deregulation of mitochondrial functions. We documented altered mitochondrial status, lowered ATP production, lowered mitochondrial mass, and changes in mitochondrial morphology (shortened and condensed mitochondria) both in melanoma and colorectal carcinoma cells. Overexpression of *TNFα* was not linked with significant affection of the subpopulation of cancer stem-like cells in vitro. However, we could demonstrate a decrease in aldehyde dehydrogenase (ALDH) activity up to 50%, which is associated with to the stemness phenotype.

**Conclusions:**

Our in vitro study of direct TNFα influence demonstrates two distinct outcomes in tumor cells of different origin, in non-epithelial malignant melanoma cells of neural crest origin, and in colorectal carcinoma cells derived from the epithelium.

**Supplementary Information:**

The online version contains supplementary material available at 10.1186/s12885-021-08237-1.

## Background

Tumor necrosis factor α (TNFα), a key inflammatory cytokine, can drive both tumor elimination and promotion in cancer patients, depending on the dose and cancer type [1–3]. Overexpression of *TNFα* in engineered tumor cells (myeloma, fibrosarcoma, breast carcinoma) blocks their engraftment and growth in mice and creates a tumor suppressive microenvironment [4–7]. The effect of TNFα is pleiotropic, modulating the tumor microenvironment via paracrine mechanisms in the context of the cancer tissue. Despite possible positive antitumor effects, the use of this cytokine for therapeutic applications in humans is very limited. TNFα was used as a ‘pre-activator’ of human mesenchymal stromal cells (MSCs), which were stimulated to produce pro-apoptotic ligand TRAIL and reduced growth of experimental metastases [8]. Engineered MSC overexpressing *TNFα*, coinjected with malignant melanoma cells, reduced growth of subcutaneous xenografts. Such engineered TNFα-MSC kept the TNFα production for more than 3 months and lost their significant tumor supportive potential [9]. But they had no inhibitory potential on induced experimental melanoma lung metastases after systemic administration [10].

The subpopulation of cancer stem-like cells (CSCs) is believed to be responsible for the relapse of cancer disease and is able to initiate tumor growth and metastases [11, 12]. CSCs are identified as the population of tumor cells positive for the unique composition of CSCs markers (CD133, CD44, CD166, ALDH positivity, and others, depending on cancer type). TNFα can affect CSCs subpopulation in vitro and enhances CSCs-like phenotype of oral squamous cell carcinoma [13] and pancreatic cancer cells [14]. Inflammatory factors can promote breast CSCs via canonical NF-κB/HIF1α signaling, enhanced by p53 inactivation [15] or via the non-canonical NF-κB pathway [16]. Long-term treatment of breast cancer cell line with a combination of TNFα and TGFβ induced epithelial-mesenchymal transition, mammosphere formation, and extreme overexpression of genes associated with stemness [17].

Between senescence and autophagy (both considered as possible hallmarks of cancer) [18] is a strong relationship and both are usually started in stress conditions, such as oxidative stress, DNA damage, mitochondrial damage and oncogene activation. Studies suggest that autophagy promotes senescence, while other reports show that mechanism of autophagy can be anti-senescent [19]. Autophagy is usually cytoprotective, maintaining healthy cells, preventing tissue damage, and cancer initiation. After suppression of the autophagy, an increase of cancer-related inflammation was observed. On the other hand, autophagy enhances stress tolerance and fulfils the high nutrient demands of cancer cells (reviewed in [20, 21]). Senescence is also a tumor-suppressive mechanism, eliminating potentially dangerous mutated cells and providing a barrier against tumorigenesis. Premature senescence is one of the outcomes of chemo- and radiotherapy in cancer treatment. Senescent cells in situ affect their microenvironment by secretion of chemokines, pro-inflammatory cytokines, and proteases, creating senescence-related secretory phenotype (SASP) (reviewed in [19, 22]). All processes initiated by TNFα are under the dominant control of ubiquitously expressed receptor TNFR1, although receptor TNFR2 may contribute to these changes as well. TNFα triggers both autophagy and pro-survival NF-κB signaling via receptor TNFR1 [23]. TNFα is directly involved in the generation of reactive oxygen species. Because TNFα is affecting mitochondrial ATP production and homeostasis, it is manifested as a mild uncoupler of the respiratory chain [24, 25]. In recent years, mitochondria of cancer cells are discussed as new possible targets for antitumor therapy, and the relationship between high mitochondrial mass and stemness phenotype is intensively studied [26, 27].

In order to study the direct effects of TNFα on tumor cell biology, we engineered cells of two different origins continually overexpressing human *TNFα* gene: epithelial colorectal adenocarcinoma lines HT29, HCT116, and malignant melanoma lines A375 and M4Beu of neural crest origin. We examined the subpopulation of CSCs, including the ALDH enzyme activity. We also aimed to show the interconnection of apoptosis, changes in mitochondrial status, autophagy and senescence accompanied by changes of mRNA expression profile.

## Methods

### Cell lines and chemicals

Human melanoma cell lines A375 (ECACC 88113005), M4Beu (kindly provided by Dr. J. Bizik, BMC SAS), human colorectal carcinoma cell lines HT29 (ECACC 91072201; authenticated by STR Profiling) and HCT116 (ATCC® CCL-247™; authenticated by STR Profiling) were maintained in high-glucose (4.5 mg/ml) Dulbecco’s modified Eagle’s medium (DMEM) or RPMI 1640 medium (Biochrom AG, Berlin, Germany). Media were supplemented with 5 or 10% fetal bovine serum (FBS) (Biochrom AG) and 2 mM glutamine. Cells were treated with recombinant human TNFα protein (PeproTech, London, UK; 100 ng/ml) for indicated time, or with zafirlukast (Sigma-Aldrich, St Louis, MO, USA; 100 μM) for 1 h. Cells were maintained in a humidified atmosphere at 37 °C and 5% CO_2_. Cell lines were regularly tested for mycoplasma contamination based on PCR.

All chemicals were purchased from Sigma-Aldrich if not stated otherwise.

### Retroviral transduction

Target cells were transduced with Moloney Murine Leukaemia virus-derived replication-deficient retroviral particles ST40hTNFa bearing human *TNFα* transgene as described previously [9]. At the multiplicity of infection (MOI) of 5–10, transduction efficiency of 50–90% was achieved. Transduction with ST40hTNFa retrovirus was verified by PCR, reverse transcriptase quantitative PCR (RTqPCR), and ELISA (Human TNFα Mini ELISA Development Kit, PeproTech). Engineered cell lines were named as A375hTNFa, M4BeuhTNFa, HT29hTNFa, and HCT116hTNFa.

### Kinetic measurement of cell proliferation and caspase 3/7 activation

Octaplicates of 3.0 × 10^3^ cells per well were seeded in 96-well plates. Cell proliferation, based on cell confluence analysis, was monitored for 6 days or until the control cells reached 100% confluency. For monitoring of apoptosis induction, the CellPlayer 96-Well Caspase 3/7 reagent (Essen BioScience, Welwyn Garden City, UK) was added to a final concentration of 5 μM. Phase contrast and fluorescent images were acquired every 3 h using the IncuCyte ZOOM Kinetic Imaging System (Essen BioScience) and four images were taken per well. Data were analyzed using IncuCyte ZOOM software. Apoptosis activation was evaluated by dividing the number of specific green fluorescent objects with activated caspase-3/7 by the percentage of the cell confluence, as described is [9]. Proliferation was expressed as mean value of cell confluence in % ± SDs.

### Immunophenotyping

For the evaluation of CSCs-like cell surface markers, the following fluorochrome-conjugated antibodies were used: CD24-PE, CD26-PE, CD44-PE/CD44-APC, CD133-PE/CD133-APC, CD271-PE, EpCAM-PE (Miltenyi Biotec, Bergisch Gladbach, Germany), CD166-PE (ALCAM, Immunotech, France), and cMET-APC (R&D Systems, Abingdon, UK). Dead cells were excluded from the analysis based on DAPI staining. Cells were analyzed by BD FACSCanto™ II flow cytometer (Beckton Dickinson, USA) equipped with FacsDiva program. FCS Express software was used for the evaluation.

### Spheroid formation assay

For 3D cultivation of spheroids, cells were seeded at a density of 3.0 × 10^3^ cells per well in ultra-low attachment 24-well plates (Corning, NY, USA). Spheroids were grown for 3–4 days in DMEM/F12 medium supplemented with 50 ng/ml EGF (Miltenyi Biotec), 20 ng/ml FGF (Miltenyi Biotec), B27 supplement (diluted 1:100, Miltenyi Biotec) and 2 mM GlutaMAX (Gibco, Thermo Fisher Scientific, Waltham, USA). Spheroids were passaged with TrypLE Reagent (Gibco) after 4 days of growth. Images were obtained using Zeiss Axiovert.A1 microscope equipped with an Axiocam 208 color camera and lengths of spheres were measured using ZEN 2 software (Zeiss, Germany).

### Monitoring of mitochondrial status, morphology, and intracellular ATP

Mitochondrial mass in tumor cells was stained with fluorescent dye MitoTracker® Deep Red FM (25 nM) (Invitrogen, Thermo Fisher Scientific, Waltham, USA; Cat. no. M22426) for 30 min at 37 °C. Mitochondrial activity/mitochondrial membrane potential was monitored with MitoTracker® Orange CMTMRos (250 nM) (Invitrogen, Cat. no. M7510) also for 30 min at 37 °C. Samples were prepared as triplicates of 2.5 × 10^5^ cells and fluorescence was measured using BD FACSCanto™ II flow cytometer (Beckton Dickinson); values are expressed as mean fluorescence intensities + SDs.

Intracellular ATP levels were measured with CellTiterGlo® Luminescent Cell Viability Assay (Promega Corporation, Madison, WI, USA) on GloMax® Discover Microplate Reader (Promega Corporation). Exponentially growing live cells were harvested and pentaplicates of 2.0 × 10^4^ cells were seeded in 96-well plates and stained immediately. Values representing ATP levels were expressed as means of relative luminescent units + SDs.

Mitochondrial morphology was evaluated by fluorescence microscopy. Cells growing on microscopic slides were stained with MitoTracker® Orange CMTMRos (100 nM) for 30 min at 37 °C, washed, fixed with 4% paraformaldehyde in PBS for 15 min at room temperature, and permeabilized with 0.05% Triton-X100 in PBS for 15 min. Nuclei were visualized by DAPI; images were analyzed by AxioImager.Z2, Metafer (Alogo, Ltd., Czech Republic) at magnification 630x and Isis upgrade software (Alogo, Ltd.).

### Monitoring of mitochondrial DNA content

Genomic and mitochondrial DNA from cultured cells was isolated together by DNazol reagent (BioTeke Corporation, Beijing, China). Mitochondrial DNA content represented by mtDNA gene for 16S rRNA was normalized to a single copy nuclear gene for β2-microglobulin using quantitative real-time PCR according Venegas et al. [28].

### Evaluation of aldehyde dehydrogenase (ALDH) activity

To determine the ALDH activity in tumor cell lines, ALDEFLUOR™ Kit (StemCell Technologies) was used according to the manufacturer’s instructions. Dead cells were excluded from the analysis based on DAPI or 7AAD staining. Cells were analyzed by BD FACSCanto™ II flow cytometer (Beckton Dickinson) equipped with FacsDiva program. FCS Express software was used for the evaluation.

### Autophagy detection

Triplicates of 1.5–3.0 × 10^3^ of cells per well were seeded in 12-well plates, incubated for 3–4 days and 1 day prior to staining, fresh media containing 10% FBS were added to the cells. Autophagic vacuoles in live cells were stained with CYTO-ID® Autophagy detection kit (Enzo Life Sciences, Farmingdale, USA) for 30 min according to the manufacturer’s instructions. Green fluorescent CYTO-ID signal in autophagic cells was monitored by BD FACSCanto™ II flow cytometer (Beckton Dickinson) or IncuCyte ZOOM Kinetic Imaging System (Essen BioScience); values represent mean fluorescent intensities + SDs.

### Senescence β-galactosidase staining

Duplicates of 5–10 × 10^3^ of cells per well were seeded in 6-well plates and incubated for 6–7 days. Recombinant human TNFα protein was added 24 or 48 h after seeding at concentration 100 ng/ml. Cells were stained with Senescence β-Galactosidase Staining Kit (Cell Signaling Technology, Danvers, USA) for 24 h according to the manufacturer’s instructions. Senescent cells containing blue precipitates were visualized by Zeiss Axiovert 40C microscope and ZEN 2 software (Zeiss, Germany).

### Analysis of gene expression

Expression analysis of mRNA for genes listed in Table 1 was determined by reverse transcriptase quantitative PCR (RT-qPCR). Total RNA was isolated from 5 × 10^5^ cells using the NucleoSpin RNA II kit (Macherey-Nagel, Dueren, Germany) and was depleted from genomic DNA by DNase treatment (DNase I, RNase-free; Thermo Fisher Scientific). Next, 2 μg of total RNA was reverse transcribed using the SensiFAST cDNA Synthesis kit (Bioline, London, UK) in one reaction. RT-qPCR was performed in triplicates or tetraplicates in Brilliant III Ultra-Fast SYBR QPCR Master Mix (Agilent Technologies, Santa Clara, USA), 250 nM concentration of primers, and 1 μl template cDNA per one reaction. The protocol for RT-qPCR was started with the activation step at 95 °C for 3 min and followed by 40 cycles of the denaturation step at 95 °C for 15 sec and annealing/polymerization at 60 °C for 15 sec with plate read step at 75 °C. PCR was performed in Bio-Rad 96FX cycler (Bio-Rad Laboratories, Hercules, USA) and analyzed with Bio-Rad CFX Manager software version 1.6 as normalized fold expression using the 2^-ΔΔCq^ method. The gene for hypoxanthine phosphoribosyl transferase 1 (*HPRT1*) was used as a reference gene. Oligonucleotides were synthesized by Metabion, Int. (Planegg, Germany) (Table 1).

**Table 1:** List of primers used for RT-qPCR

Gene	Forward primer sequence 5′ → 3′	Reverse primer sequence 5′ → 3′	Amplicon length (bp)
*TNFaRT*	CAGAGGGAAGAGTTCCCCAG	CCTTGGTCTGGTAGGAGACG	325
*TNFR1*	ACCAAGTGCCACAAAGGAAC	GTTTTCTGAAGCGGTGAAGG	105
*TNFR2*	GCTCTGACCAGGTGGAAACTCAAG	GGATGAAGTCGTGTTGGAGAACG	224
*TRAIL*	ACCAACGAGCTGAAGCAGAT	ACGGAGTTGCCACTTGACTT	141
*IL6*	AAAGAGGCACTGGCAGAAAA	TTTCACCAGGCAAGTCTCCT	99
*CD133*	TTGTGGCAAATCACCAGGTA	TCAGATCTGTGAACGCCTTG	162
*Nestin*	AGCCCTGACCACTCCAGTTTAG	CCCTCTATGGCTGTTTCTTTCTCT	128
*OCT4*	ACATCAAAGCTCTGCAGAAAGAACT	CTGAATACCTTCCCAAATAGAACCC	127
*SOX2*	GGAAAGTTGGGATCGAACAA	GCGAACCATCTCTGTGGTCT	145
*WNT7B*	GTCCTGTACGTGAAGCTCGG	GTACTGGCACTCGTTGATGC	174
*STAT3*	GCCAGAGAGCCAGGAGCA	GGTTCAGCACCTTCACCATT	174
*HPRT1*	GACCAGTCAACAGGGGACAT	CCTGACCAAGGAAAGCAAAG	132

### Statistical analysis

The results are expressed as the means + SDs (flow cytometry data, RTqPCR) or SEM (RTqPCR Fig. 6). Values were tested for normality and then compared using a two-tailed unpaired Student’s t-test or nonparametric Mann-Whitney U test in GraphPad Prism program, version 6 (GraphPad Software Inc., San Diego, CA, USA); *p < 0.05, **p < 0.01, ***p < 0.001 and ****p < 0.0001 were considered statistically significant.

## Results

### Overexpression of *TNFα* has a mild cytotoxic effect on tumor cells in vitro and is limited by the availability of TNFR1 and TNFR2 receptors

**Fig. 1:**
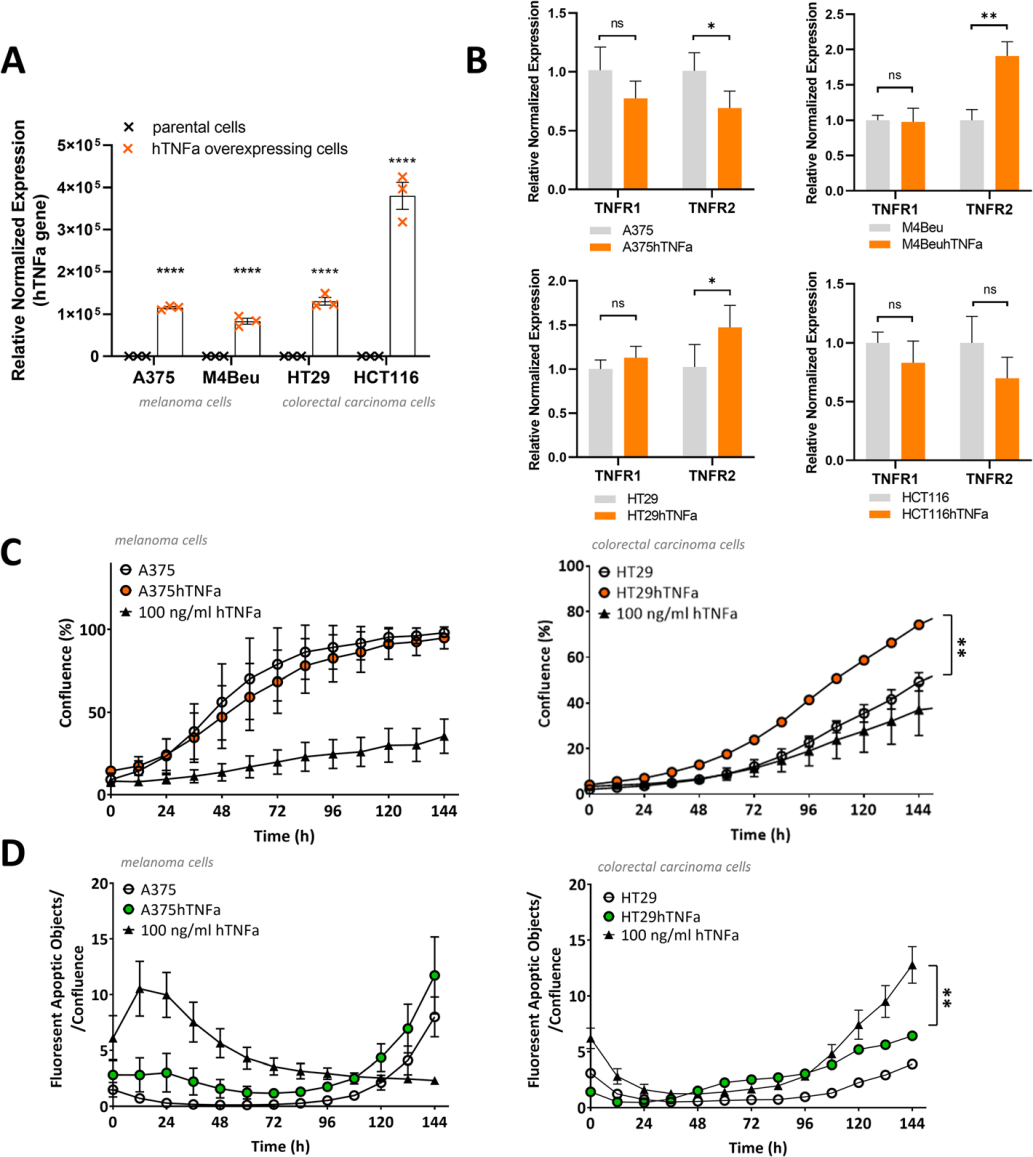
Extensive mRNA overexpression of *TNFα* gene in engineered tumor cells does not change significantly the expression of main receptor TNFR1 and did not decrease the ability to proliferate. Cells can overcome slight caspase induction. Reverse transcriptase quantitative PCR for *TNFα* gene (**a**) and receptors TNFR1, TNFR2 (**b**); expression was normalized to the HPRT reference gene, values are means of triplicates or quadruplicates ± SDs; unpaired t-test was used to statistical analysis. **c** Continual monitoring of cell proliferation; IncuCyte ZOOM Kinetic Imaging System; means of octaplicates ± SDs; unpaired t-test was used to statistical analysis of confluence at hour 144. **d** Specific apoptosis induction monitoring (caspase 3/7 activation); IncuCyte ZOOM Kinetic Imaging System; means of octaplicates ± SDs; 100 ng/ml hTNFa – parental cells treated with recombinant human TNFα protein. A375, M4Beu – melanoma cells, HT29, HCT116 – colorectal carcinoma cells

In engineered melanoma cells (A375hTNFa, M4BeuhTNFa) and colorectal carcinoma cells (HT29hTNFa, HCT116hTNFa), stable retroviral transduction elevated the mRNA expression of *TNFα* transgene up to 130.000x (engineered cells with approx. Cq 14–16 vs. parental cells with Cq 30–33; RTqPCR, Fig. 1a). Naïve parental cell lines displayed low basal mRNA expression of the intrinsic *TNFα* gene. ELISA confirmed concentrations of 50–150 ng/ml of soluble TNFα protein secreted by engineered cells reaching confluence of 90–100% into the media during 24 h, while parental cells displayed low or no TNFα protein secretion (Additional file 1). Expression of the crucial receptor TNFR1 was not changed significantly, but the receptor TNFR2 was down- or upregulated in *TNFα* overexpressing cells with fold change 0.75–2.0 (Fig. 1b).

The viability and proliferation rate of the cells were not decreased (Fig. 1c), but HT29hTNFa cells displayed an accelerated growth rate, similar to other retrovirally transduced cells. Specific executioner caspase 3/7 activation (Fig. 1d) was not robust. In fast proliferating melanoma cells A375hTNFa, the onset of apoptosis was detected during the first 48 h, in HT29hTNFa carcinoma cells, apoptosis was induced on day 3–4 of cultivation. Engineered tumor cells tolerated extensive *TNFα* overexpression well and easily overcame partial apoptosis induction.

### Autophagy and senescence are induced in malignant melanoma cell lines overexpressing *TNFα*

**Fig. 2:**
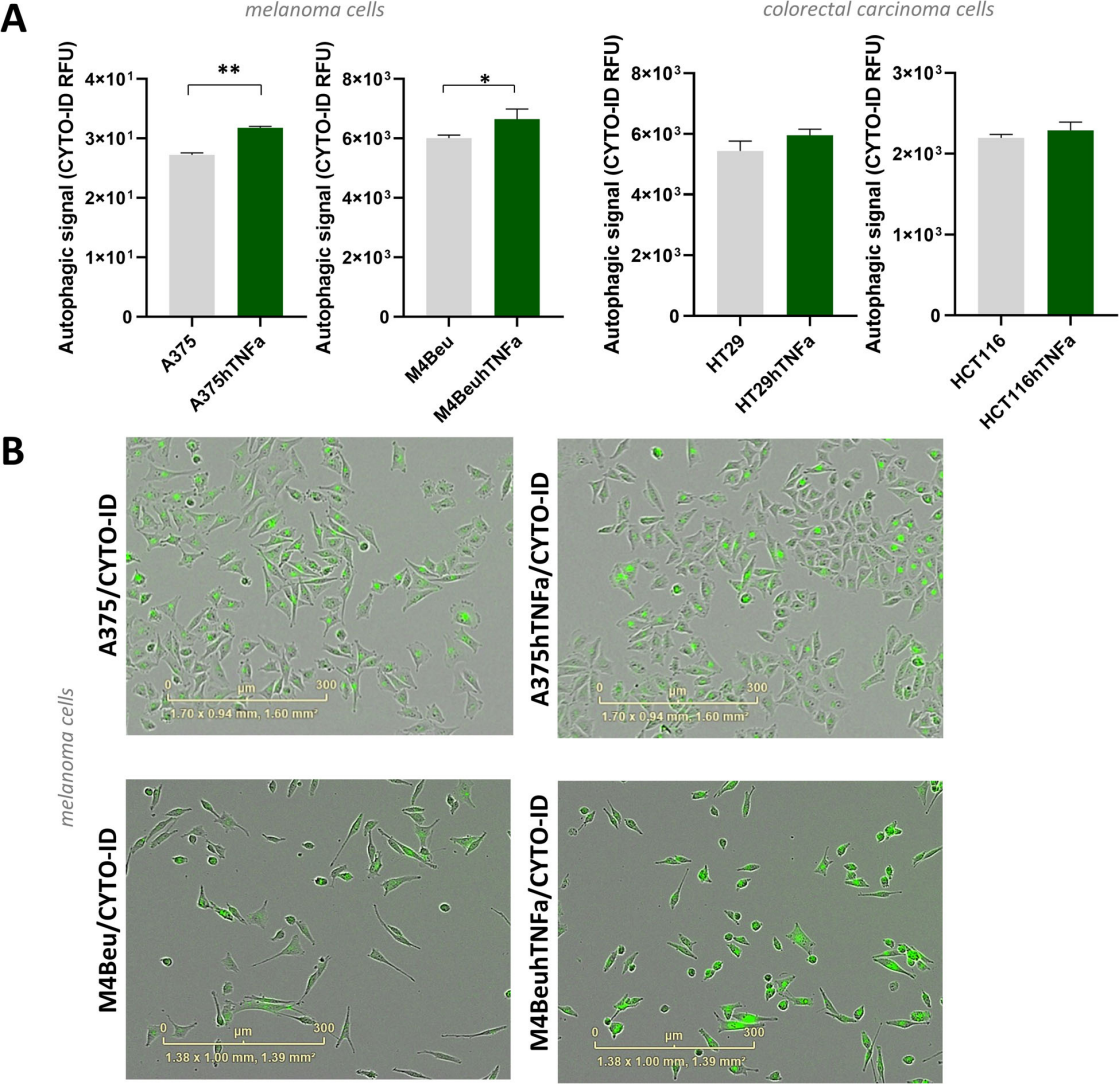
Autophagy is induced in engineered melanoma cells A375hTNFa and M4BeuhTNFa. **a** Detection of autophagic flux by CYTO-ID autophagy detection reagent; flow cytometry, means of triplicates + SDs, unpaired t-test was used to statistical analysis, *p < 0.05. **b** Representative images of green fluorescent autophagic cells stained with CYTO-ID; fluorescent/light-microscope images from IncuCyte ZOOM Kinetic Imaging System, scale bar: 300 μm

Autophagy was monitored by labeling with CYTO-ID dye specific for autophagic vacuoles (Fig. 2). In A375hTNFa cells, an increase of autophagic objects of 17% was observed on day 3 or 4 of cultivation. Increase of 10% was detected also in other melanoma cell line M4BeuhTNFa. Colorectal cancer cells displayed no significant increase of autophagic objects (Fig. 2a), but the addition of recombinant human TNFα protein in concentration 100 ng/ml increased autophagy in HT29 cells (data not shown).

**Fig. 3:**
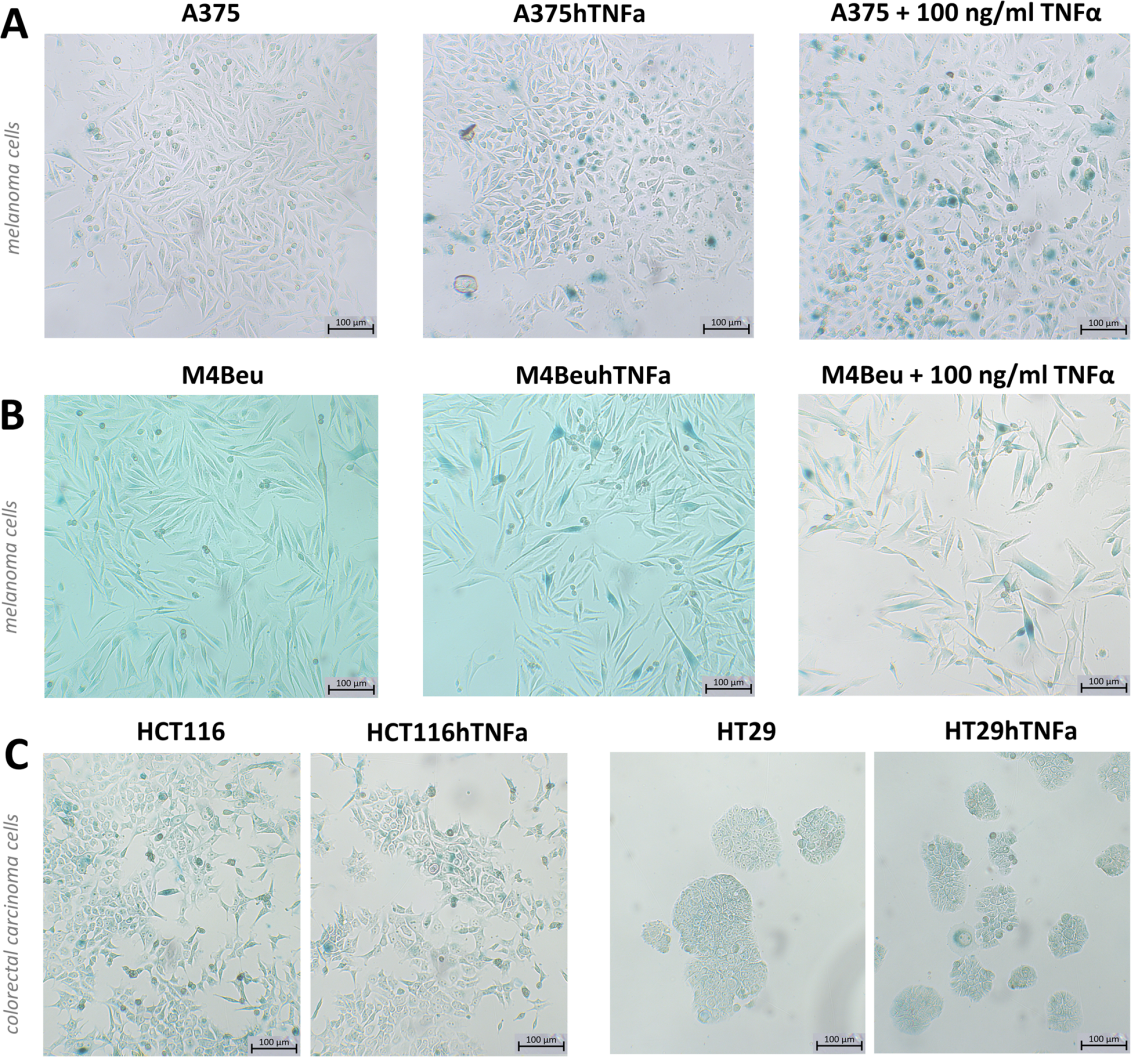
Senescence induction in engineered melanoma cells overexpressing *TNFα*. Melanoma cells A375hTNFa (**a**, middle), M4BeuhTNFa (**b**, middle) entering senescence and senescent parental melanoma cells after treatment with recombinant TNFα (100 ng/ml) (**a**, **b**, left panel). In colorectal carcinoma cells HT29hTNFa and HCT116hTNFa (**c**) senescence is not induced. Senescent cells are blue enlarged cells; β-galactosidase staining on day 6 of cultivation; light microscope images, scale bar: 100 μm

Significant senescence induction was confirmed in melanoma lines overexpressing *TNFα* on days 6 and 7. Characteristic enlarged senescent cells containing blue-dyed precipitates indicating increased senescence-associated β-galactosidase activity appeared in both melanoma lines A375hTNFa and M4BeuhTNFa (Fig. 3a-b). Senescence induction was observed also in parental melanoma cells treated with recombinant human TNFα protein (Fig. 3a-b). In colorectal carcinoma cells HT29hTNFa and HCT116hTNFa, no differences in the incidence of senescent cells were observed. HCT116 parental cells entered senescence spontaneously and more frequently in comparison to the HT29 cells (Fig. 3c).

### Mitochondrial functions of tumor cells are affected by *TNFα* overexpression

**Fig. 4:**
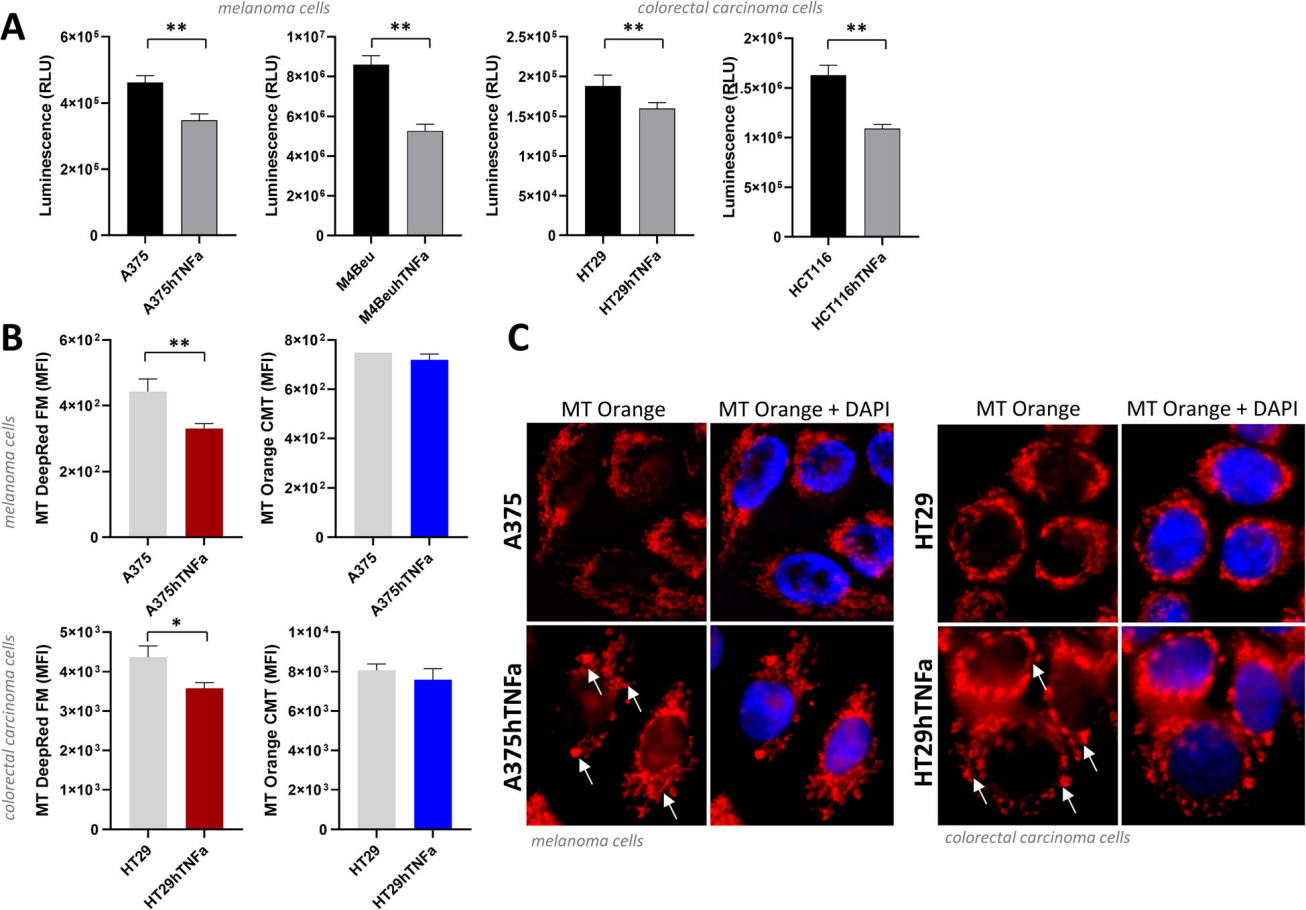
Decrease of mitochondrial mass and intracellular ATP is accompanied with morphological changes of mitochondria in tumor cell lines overexpressing *TNFα*. **a** Measurement of intracellular ATP (Luminescent Cell Viability Assay, Promega) in exponentially growing cells; means of pentaplicates + SDs, unpaired t-test was used to statistical analysis. **b** Staining of mitochondrial mass with MitoTracker DeepRedFM (25 nM) (left) and detection of mitochondrial activity with MitoTracker OrangeCMT (250 nM) (right) in cells cultivated for 3–4 days; flow cytometry, values are expressed as the means of triplicates + SDs, unpaired t-test was used to statistical analysis. **c** Fluorescence microscopy of mitochondrial morphology in adherent cells, red – mitochondria stained with Mitotracker OrangeCMT, blue – nuclei/DAPI; white arrows – condensed/shortened mitochondria in hTNFα overexpressing cells; magnification 630x

Because TNFα is manifested as a mild uncoupler of oxidative phosphorylation, the mitochondrial status of engineered tumor cells was monitored. Cellular ATP content was found to be reduced to 75, 61, 85 and 67% in all analyzed *TNFα*-overexpressing cell lines (Fig. 4a). Mitochondrial mass stained with MitoTracker DeepRed was significantly reduced in A375hTNFa cells (to 75%) and in HT29hTNFa cells (to 82%) compared to parental cells (Fig. 4b). In two other tested cell lines, M4BeuhTNFa and HCT116hTNFa, mitochondrial mass maintained stable. Mitochondrial membrane potential specifically stained with MitoTracker Orange CMT, indicating mitochondrial activity, remained unchanged in engineered cells. Quantitative real-time PCR revealed that the cells also kept constant mitochondrial DNA content under the *TNFα* overexpression (Additional file 2). Mitochondrial morphology and mitochondrial activity visualized by MitoTracker Orange CMT was not changed significantly, but more condensed and shortened mitochondria with perinuclear localization seemed to be more abundant in *TNFα* overexpressing cells. Parental cells displayed more tubular mitochondrial network (Fig. 4c).

### Aldehyde dehydrogenase activity is significantly reduced in *TNFα* overexpressing cells

**Fig. 5:**
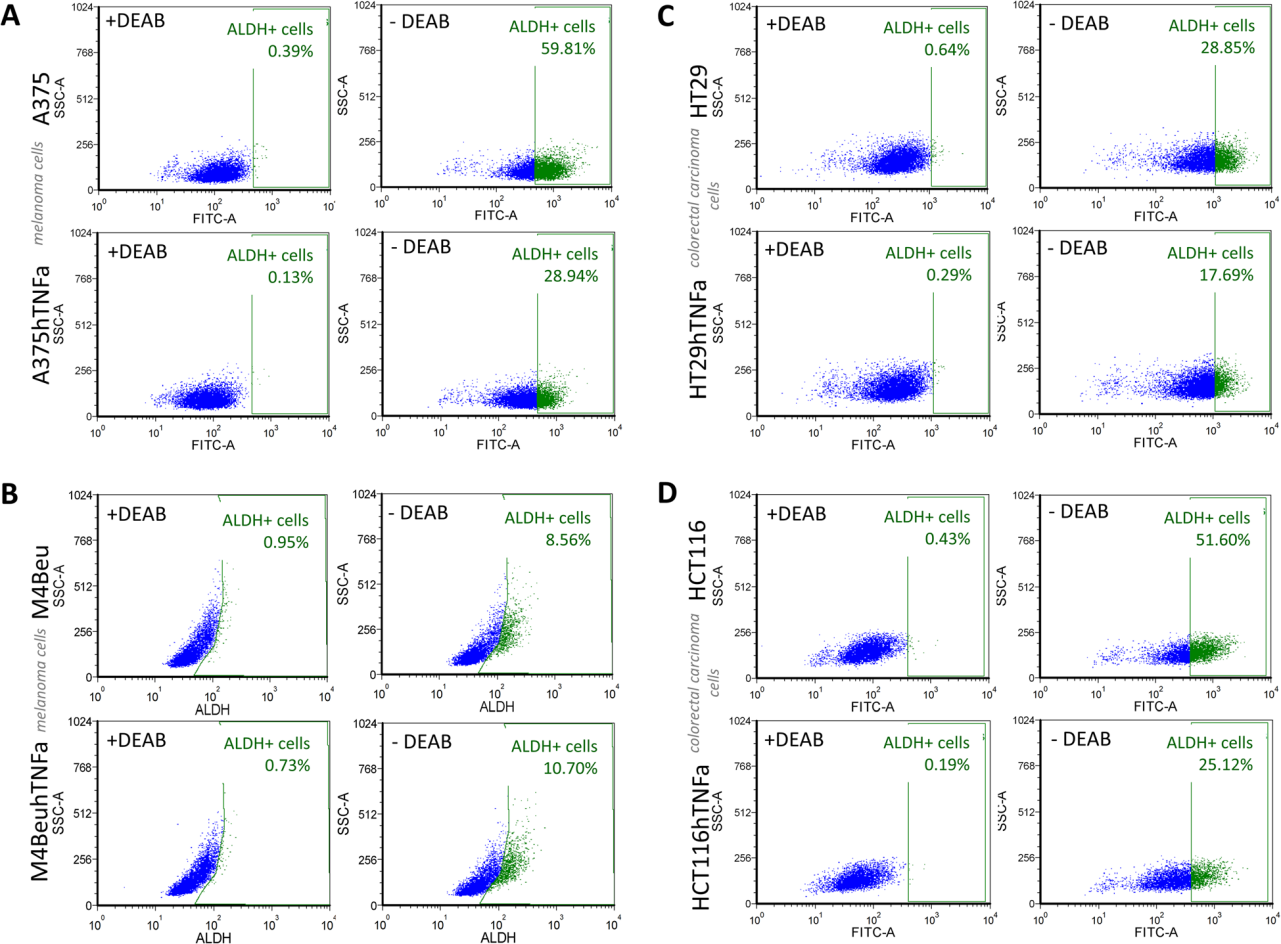
Aldehyde dehydrogenase activity (ALDH) is reduced in *TNFα* overexpressing tumor cells. Proportion of ALDH positive (ALDH+) cells in engineered (**a**) melanoma (A375hTNFa) and (**c**, **d**) colorectal cancer (HT29hTNFa, HCT116hTNFa) cell lines decreases approx. by a half in comparison to parental cells; (**b**) M4Beu cells have low innate ALDH activity and increased proportion of ALDH+ cells. Flow cytometry, ALDEFLUOR assay

High expression of aldehyde dehydrogenase (ALDH) has been reported in both normal and cancer stem cells and ALDH is considered as a marker of stem cells. The proportion of aldehyde dehydrogenase positive cells (ALDH+) under the overexpression of *TNFα* in tumor cells was reduced approximately to 50–60% in comparison to parental cells (Fig. 5) in three engineered cell lines. Melanoma cells M4Beu displayed low innate ALDH activity (8.5% of ALDH+ cells), increasing to 10.7% in engineered cells M4BeuhTNFa. Treatment with inhibitor zafirlukast, a compound stabilizing non-functional conformational state of TNFR1 [29], returned the ALDH activity level in engineered melanoma cells A375hTNFa to the level of ALDH activity in parental cells (83.5% vs. 85.4% of ALDH+ cells, unpublished data). In HT29 cells, no significant differences were monitored. Treatment with inhibitor of TNFR1 followed by treatment with recombinant TNFα (100 ng/ml) did not show significantly increased or decreased levels of ALDH activity in A375 and HT29 cells (unpublished results).

### mRNA expression profile shows changes in expression of apoptosis-inducing agent TRAIL and cytokine IL6. CSCs- like markers remained unchanged

**Fig. 6:**
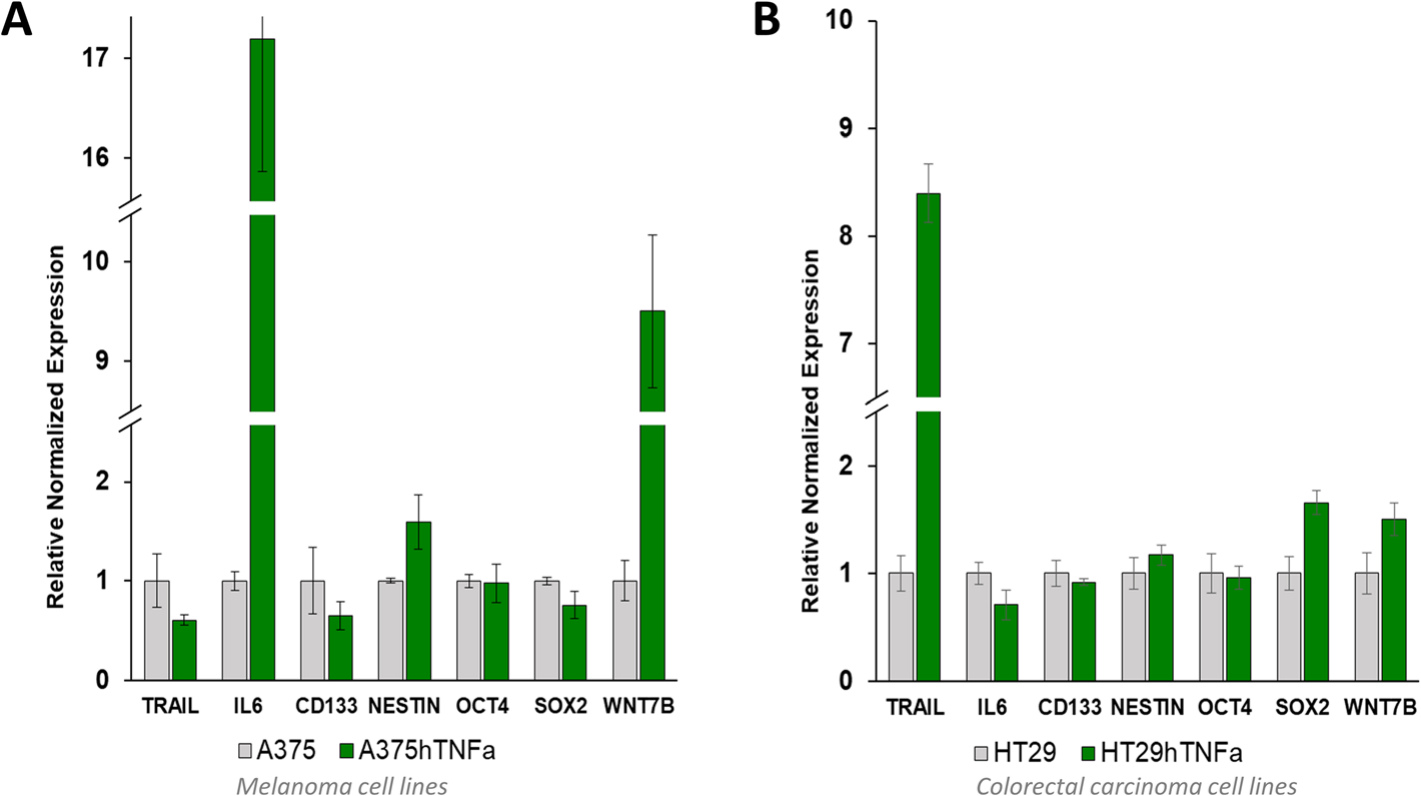
Expression profile changes in engineered hTNFα tumor cells. **a** In melanoma cells A375hTNFa, mRNA of inflammatory cytokine *IL6* is extensively overexpressed; (**b**) carcinoma cells HT29hTNFa are stimulated to overexpress TNFα apoptosis-inducing ligand *TRAIL* mRNA. Expression of pluripotency and stemness markers *OCT4*, *SOX2*, *NESTIN* and marker of cancer stem cells *CD133* are not changed significantly; reverse transcriptase quantitative PCR, triplicates ± SEMs

In engineered melanoma cells A375hTNFa, significant overexpression of inflammatory cytokine IL6 was induced (up to 17 times; Fig. 6a). Overexpression of *TNFα* in cells HT29hTNFa significantly stimulated expression of apoptosis-inducing ligand TRAIL (up to 8 times; Fig. 6b). In A375hTNFa and HT29hTNFa cells, expressions of selected genes encoding stemness markers *OCT4*, *SOX2*, *NESTIN*, and *CD133* were not changed significantly in comparison to the parental cells (Fig. 6). No significant changes in expression profile were detected in cells M4BeuhTNFa and HCT116hTNFa (data not shown). Only mRNA for *WNT7B* gene of WNT- signaling pathway was overexpressed in A375hTNFa cells (Fig. 6a).

Flow cytometric analysis of CSC markers (CD24, CD26, CD44, CD133, CD166, CD271, cMET, EpCAM) did not reveal significant changes in *TNFα*- engineered cells (Additional file 3). Small divergences were observed, such as a decrease of CD166 positive cells and an increase in CD24 and CD26 in HT29hTNFa carcinoma cells. Insignificant deviations in markers CD271, CD44, and CD166 were noticed in A375hTNFa, while CD133 and c-MET markers remained unchanged.

Subpopulations of CSCs are able to form 3D spheroids. Three of our four tested cell lines overexpressing *TNFα* (A375, HT29, HCT116) were able to form melanospheres or colonospheres in similar appearance, quantity, and size as their parental counterparts (Additional file 4). However, melanoma cells M4Beu were not able to grow in 3D spheres in given conditions [30].

The specific role of TNFα in stimulation the *TRAIL* or *IL6* gene overexpression was confirmed by exposing cells to TNFR1 inhibitor zafirlukast. Inhibition of TNFR1 by this component resulted in a progressive significant decrease of TRAIL expression in colorectal carcinoma cells HT29hTNFa 2 and 4 h post-treatment (Additional file 5a). In parental melanoma cells A375, zafirlukast itself, surprisingly, dramatically increased expression of *TNFα* mRNA (up to 100 times) and also increased IL6 expression (Additional file 5c-e). Its inhibitory effect was observed firstly 4 h post-treatmentand was less evident in A375hTNFa melanoma cells in comparison to colorectal carcinoma cells HT29hTNFa.

## Discussion

One of our major findings is that the overexpression of *TNFα* in engineered colorectal carcinoma and melanoma cell lines is not linked with significant affection of the subpopulation of CSCs in vitro. Monitored CSCs-surface markers, associated with stemness, resistance, tumor progression, adhesion, and invasion, were find to be not significantly up- or downregulated and TNFα makes cells only slightly more positive for these markers (CD166 and CD271 in melanoma; CD24 and CD26 in carcinoma) (Additional file 3). According to works of Storci et al. and Liu et al. [15, 16], treatment of tumor cells with TNFα increased CSCs subpopulation in breast carcinoma cell line (i.e. CD44+, ALDH+ cells, and mammosphere counts). Our findings did not show similar changes in melanoma and colorectal carcinoma cells and the mRNA expression of CSCs-related genes (*OCT4, SOX2, NANOG, CD133*) was not significantly changed. We can also demonstrate unchanged ability to grow in 3D cultures and form in vitromelanospheres and colonospheres under the *TNFα* overexpression in vitro. The ability to generate spheres in suspension culture is attributed to CSCs-subpopulation, known also as tumor-initiating cells, which are responsible for tumor formation and relapse. We can conclude, that *TNFα* overexpression in our cancer cells was not able to hit CSCs subpopulation in vitro and is not responsible for the loss of tumorigenic potential of engineered cells in vivo.

However, we can demonstrate a significant decrease in ALDH activity in vitro. Aldehyde dehydrogenase ALDH is a critical component of the oxidative stress response and detoxification, overexpressed in various tumors, promoting chemoresistance, and considered as an important CSCs marker [31]. We observed a decrease in ALDH activity up to 50%, independently of the fact, that TNFα increases ROS production. This is in contrast to the findings of Moreb et al. [32] and Liu et al. [16], showing increased both ALDH mRNA and ALDH protein activity in human bone marrow cells and malignant cells treated with TNFα, respectively. Based on our experiments with TNFR1 inhibitor zafirlukast, we can conclude that ALDH activity is (i) not affected directly by addition of recombinant TNFα protein, but (ii) in engineered cells overexpressing *TNFα*, the ALDH decrease is probably caused indirectly by unknown mechanism. Despite decreased ALDH positivity, flow cytometry measurements, and spheroid formation assay did not indicate affected subpopulation of CSCs. The issue of decreased ALDH activity under the stable overexpression of *TNFα* needs deeper investigation because there are only a few published studies till today.

Under the *TNFα* overexpression, the mRNA of two important genes was significantly upregulated: pro-inflammatory cytokine IL6 in melanoma cells A375hTNFa, and pro-apoptotic ligand TRAIL in carcinoma cells HT29hTNFa. Overexpression of *TRAIL* gene in carcinoma cells explains the increase of caspase induction in HT29hTNFa in vitro. In melanoma cells, *TNFα* overexpression accompanied by the secretion of potent cytokine IL6 will be probably able to induce a strong inflammatory response in vivo. Since IL6 increases melanoma invasiveness and elevated levels of IL6 are linked with worse prognosis in non-responding patients [33, 34], resulting effect of *TNFα* overexpression can be also adverse.

**Fig. 7:**
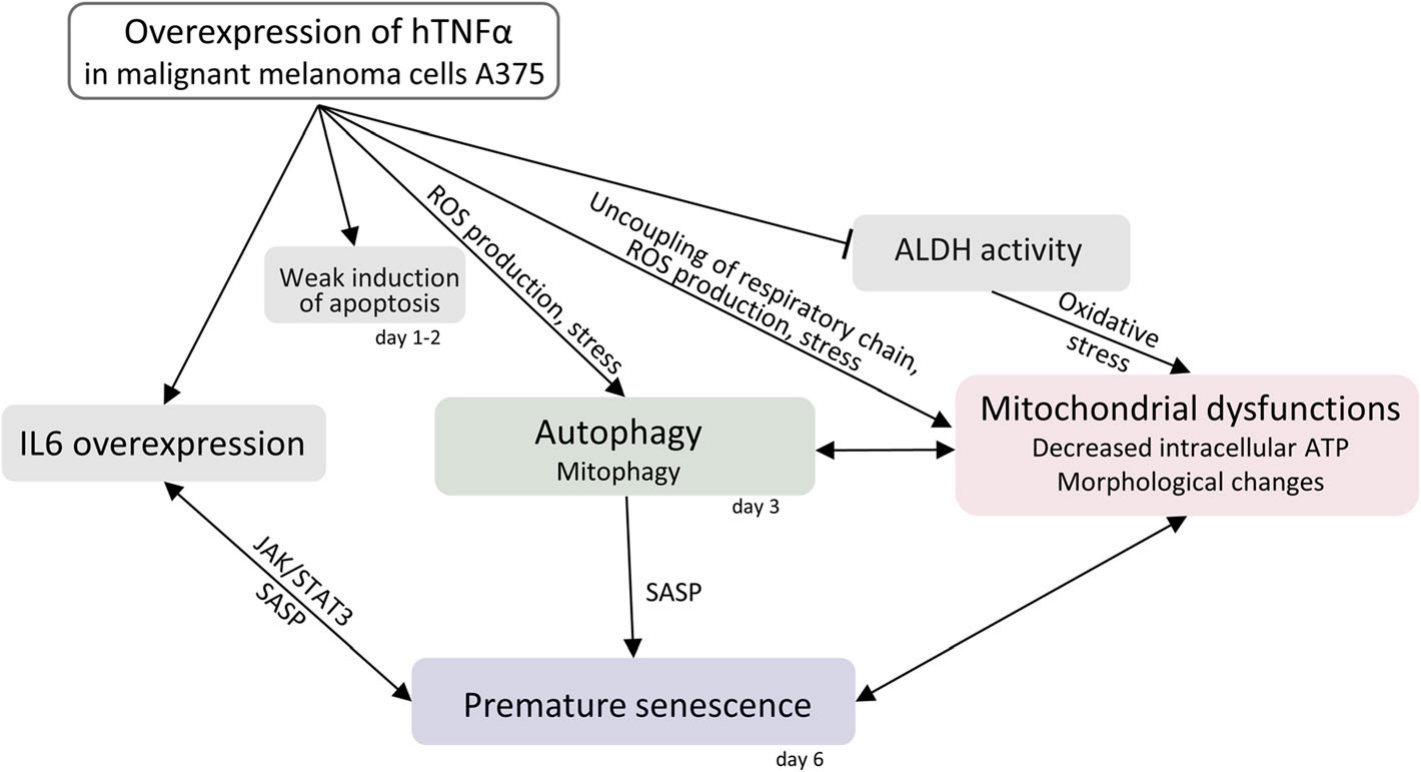
Possible relationships between *TNFα* overexpression, apoptosis, autophagy, mitochondrial status and senescence in engineered melanoma cells A375hTNFa. Overexpression of *TNFα* induces weak apoptosis activation on day 1–2 and most of the cells enter survival/inflammation pathway. TNFα induces ROS production, stress and fragmentation of mitochondria. It is manifested as a mild uncoupler and causes decreased levels of intracellular ATP. Autophagy, and probably also mitophagy, are activated on day 3 and can bring mitochondrial dysfunctions too. And mitochondrial dysfunctions can be a cause of mitophagy. ALDH activity is reduced to 50% and probably cannot ensure complete detoxification of products of oxidative stress. Overexpression of *TNFα* induces significant overexpression of *IL6,* known inducer of JAK/STAT3 signalling pathway, inducer of senescence and component of SASP. Finally, autophagy, IL6, mitochondrial dysfunctions and oxidative stress caused by TNFα lead to premature senescence in malignant melanoma cells A375hTNFa. *IL6* – interleukin 6, ALDH – aldehyde dehydrogenase, ROS – reactive oxygen species, SASP – senescence associated phenotype, JAK/STAT3 – signalling pathway

In our study, engineered malignant melanoma lines A375hTNFa and M4BeuhTNFa have displayed increased autophagy on day 3, followed by premature senescence on day 6. Both processes might be interconnected [19], following earlier apoptosis induction and deregulation of mitochondrial functions (Fig. 7). These processes can have a positive tumor-suppressive effect. It is known, that TNFα triggers premature senescence in various cell types, such as endothelial, epithelial, and leukemic cells [35, 36] and here we can confirm the senescence induction also in malignant melanoma cells. Autophagy activation after TNFα treatment was previously observed in osteoblasts [37]. However, the senescence was not induced in colorectal carcinoma cells HT29hTNFa and HCT116hTNFa suggesting the two different mechanisms of TNFα action in carcinoma cells and in melanoma cells (Fig. 7). Overexpression of *IL6* can also represent an explanation for senescence induction in melanoma because IL6 is a known initiator of senescence [38] and an important component of SASP [39, 40]. On the other hand, significant upregulation of the *TRAIL* gene was reported also in human MSCs treated with TNFα. These MSCs exerted tumor-suppressive properties on breast cancer xenografts [8].

Different mitochondrial metabolism of cancer cells, known as the Warburg effect, distinguishes tumor cells from normal cells [41] and is considered as possible target of anticancer therapy. We documented altered mitochondrial status of malignant cells under the long term overexpression of *TNFα*: lowered ATP production and lowered mitochondrial mass. This is in accordance with several studies [24, 25] showing altered mitochondrial metabolism and increased mitochondrial fragmentation in liver cells and adipocytes after TNFα treatment. The mitochondrial membrane potential of engineered *TNFα*- overexpressing cells remained unchanged in our study. Since the level of the mitochondrial mass is decreased in *TNFα-* tumor cells, we can also speculate, that there can be some increase of mitochondrial activity. Depending on energetic needs and stress stimuli, also mitochondrial morphology is continuously changing. Cancer cells display more shortened mitochondria than normal cells and the fragmentation is also more apparent after stress stimuli [42]. A study of Maeda et al. describes increased mitochondrial fission/fragmentation and release of intact mitochondria from the TNFα- treated human leukemia cells during programmed necrosis (necroptosis) and sterile inflammation [43]. In our *TNFα-* engineered cells, we have demonstrated similar altered mitochondrial morphology – more rounded and condensed mitochondria and less visible tubular network in comparison to their parental counterpart.

## Conclusions

Our work reports the impact of TNFα on cancer cells of different origin – non-epithelial malignant melanoma cells of neural crest origin and colorectal carcinoma cell lines of epithelial origin. TNFα acts differently in these cells, confirming its pluripotency and indicating two possible ways of action. In melanoma cells, inflammation/survival pathway is supported by *IL6* overexpression, and activation of autophagy followed by premature senescence is initiated. Deregulation of mitochondria and changes in mitochondrial morphology were noticed in both cancer cell types. Carcinoma cells are stimulated to overexpress pro-apoptotic gene *TRAIL* and they avoid to activate other inflammatory mediators. But the final effect of TNFα is mild in vitro*,* limited by the availability of its receptors TNFR1 and TNFR2 and presumably predominant activation of pro-survival pathway NF-κB. Despite such high overexpression of TNFα protein in engineered tumor cells, the subpopulation of CSCs responsible for tumor growth initiation remained unaffected, not increased or decreased, and is not directly linked with the loss of tumorigenic potential.

## Supplementary Information

**Additional file 1: Figure S1.** Engineered melanoma (A375hTNFa, M4BeuhTNFa) and colorectal carcinoma cells (HT29hTNFa, HCT116hTNFa) secrete high levels of TNFα protein. ELISA quantification of TNFα protein in conditioned media harvested from cells reaching 90–100% confluence during 24 h; means of triplicates + SDs.

**Additional file 2: Figure S2.** Mitochondrial DNA content is not changed in *TNFα* overexpressing melanoma (a) and colorectal carcinoma cells (b). Mitochondrial DNA content (mtDNA) represented by gene for 16S rRNA was normalized to a nuclear DNA (nDNA) represented by β2-microglobulin gene; quantitative PCR, triplicates ± SDs.

**Additional file 3: Figure S3.** Cancer stem cell-related markers remained unchanged under the overexpression of *TNFα* gene in melanoma (a) and colorectal carcinoma cells (b, c). Flow cytometry analysis; cells overexpressing *TNFα* (red line) and parental cells (black line) stained with specific anti-human antibodies against CSCs markers; isotype control antibodies as staining controls of *TNFα* overexpressing cells (blue line) and parental cells (grey line).

**Additional file 4: Figure S4.** Spheroid formation ability is not affected under the *TNFα* overexpression. Spheroids of melanoma cells A375 (melanospheres) were grown in passage 0 for 4 days; spheroids of colorectal carcinoma cells HT29 and HCT116 (colonospheres) were grown in passage 1 for 3 days. Colonospheres later quickly fused together into big spheres with length of more than 600 μm. (a) Lengths of spheres; bars represent medians, *n* = 20–30, Mann-Whitney test; (b) representative light microscope images, scale bar: 100 μm.

**Additional file 5: Figure S5.** Inhibitor of TNFR1 receptor zafirlukast (ZAF) inhibits (a) *TRAIL* gene overexpression in engineered colorectal carcinoma cells HT29hTNFa and (d) *IL6* gene expression in malignant melanoma cells A375hTNFa (highlighted with red line). (e) Zafirlukast itself also significantly increases *TNFα* gene expression in parental cells A375, so its inhibitory effect on *IL6* overexpression (d) is evident firstly after 4 h post-treatment. (a, c, d) Overexpression of *TRAIL* and *IL6* confirmed in *TNFα* overexpressing cells and in control cells treated with recombinant TNFα (100 ng/ml). Cells were treated with zafirlukast (100 μM) for 1 h at 37 °C; recombinant TNFα (100 ng/ml) was then added to the control samples for 2 and 4 h (2 h, 4 h) post- zafirlukast treatment and then all cells were immediately harvested for reverse quantitative PCR for *TRAIL*, *IL6* and *TNFα* gene. Expression normalized to *HPRT1* reference gene; values are mean of triplicates ± SDs; unpaired t-test was used to statistical analysis. Pictures (c) and (d) represent independent experiments.

## Data Availability

Datasets of this study are available through the corresponding author on reasonable request.

## Abbreviations

ALDH: Aldehyde dehydrogenase
ATP: Adenosine triphosphate
IL6: Interleukin 6
TNFα: Tumor necrosis factor α
TNFR1/TNFR2: TNF receptor 1/TNF receptor 2
TRAIL: TNF-related apoptosis-inducing ligand
MSC: Mesenchymal stromal cells
CSCs: Cancer stem-like cells
SASP: Senescence-related secretory phenotype
ROS: Reactive oxygen species
