# Supplementary figures and images for: Overexpression of TNFα induces senescence, autophagy and mitochondrial dysfunctions in melanoma cells
Source: BMC Cancer. 2021 May 6;21:507. doi: 10.1186/s12885-021-08237-1 (PMC8101174; doi:10.1186/s12885-021-08237-1)

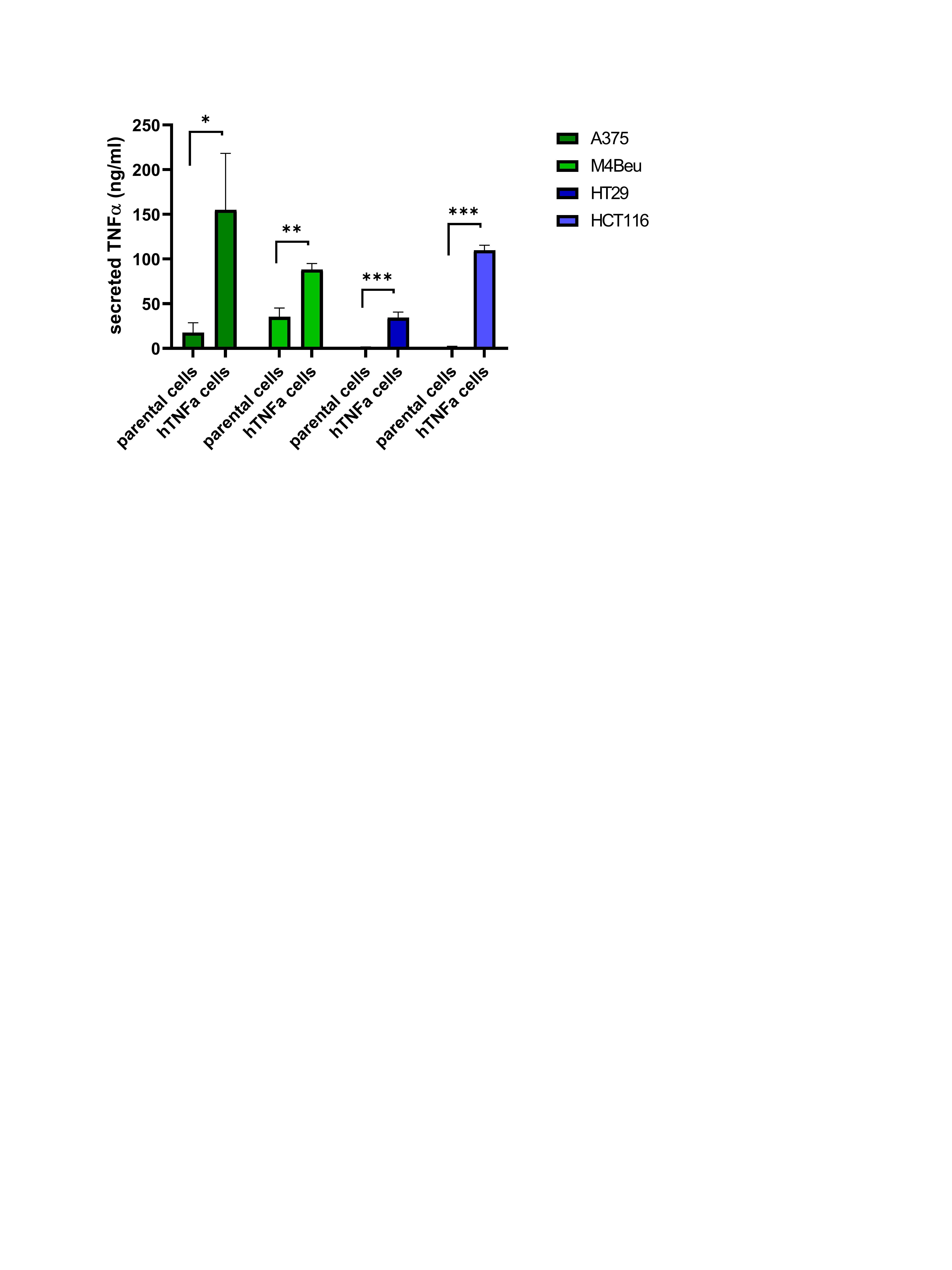

Supplement: Supplementary file 1 — Additional file 1: Figure S1. Engineered melanoma (A375hTNFa, M4BeuhTNFa) and colorectal carcinoma cells (HT29hTNFa, HCT116hTNFa) secrete high levels of TNFα protein. ELISA quantification of TNFα protein in conditioned media harvested from cells reaching 90–100% confluence during 24 h; means of triplicates + SDs. [file 12885_2021_8237_MOESM1_ESM.tif]

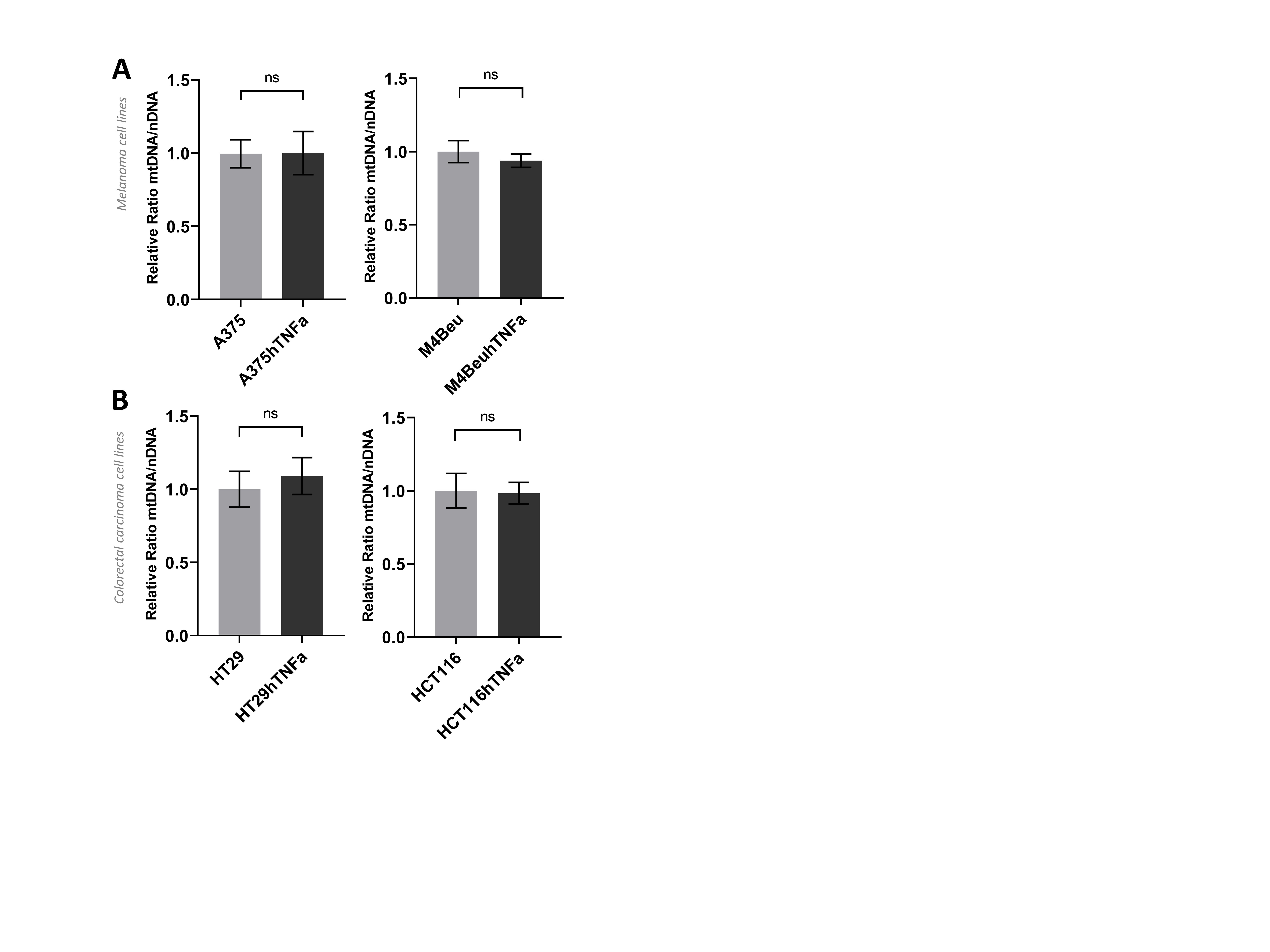

Supplement: Supplementary file 2 — Additional file 2: Figure S2. Mitochondrial DNA content is not changed in TNFα overexpressing melanoma (a) and colorectal carcinoma cells (b). Mitochondrial DNA content (mtDNA) represented by gene for 16S rRNA was normalized to a nuclear DNA (nDNA) represented by β2-microglobulin gene; quantitative PCR, triplicates ± SDs. [file 12885_2021_8237_MOESM2_ESM.jpg]

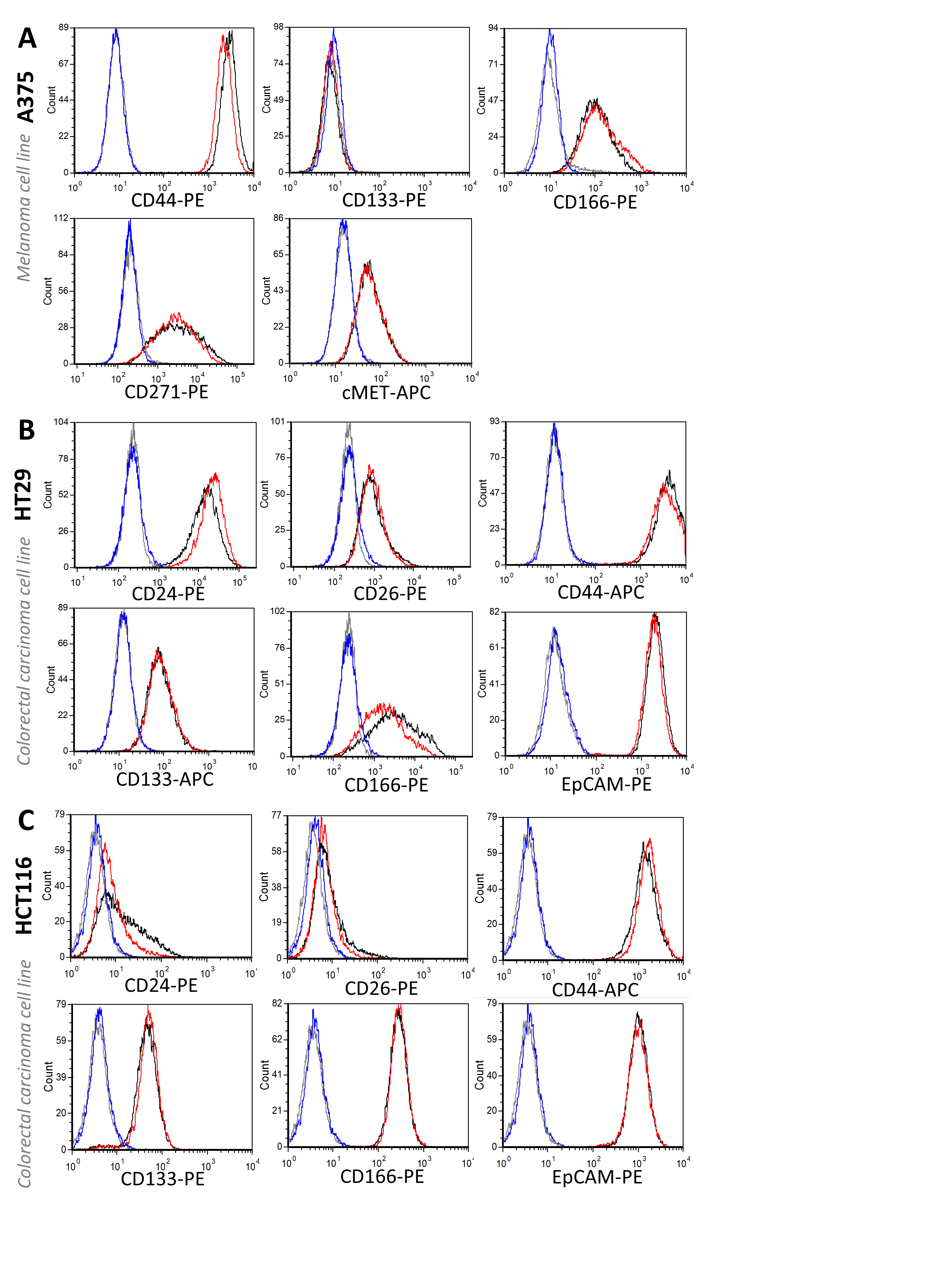

Supplement: Supplementary file 3 — Additional file 3: Figure S3. Cancer stem cell-related markers remained unchanged under the overexpression of TNFα gene in melanoma (a) and colorectal carcinoma cells (b, c). Flow cytometry analysis; cells overexpressing TNFα (red line) and parental cells (black line) stained with specific anti-human antibodies against CSCs markers; isotype control antibodies as staining controls of TNFα overexpressing cells (blue line) and parental cells (grey line). [file 12885_2021_8237_MOESM3_ESM.tif]

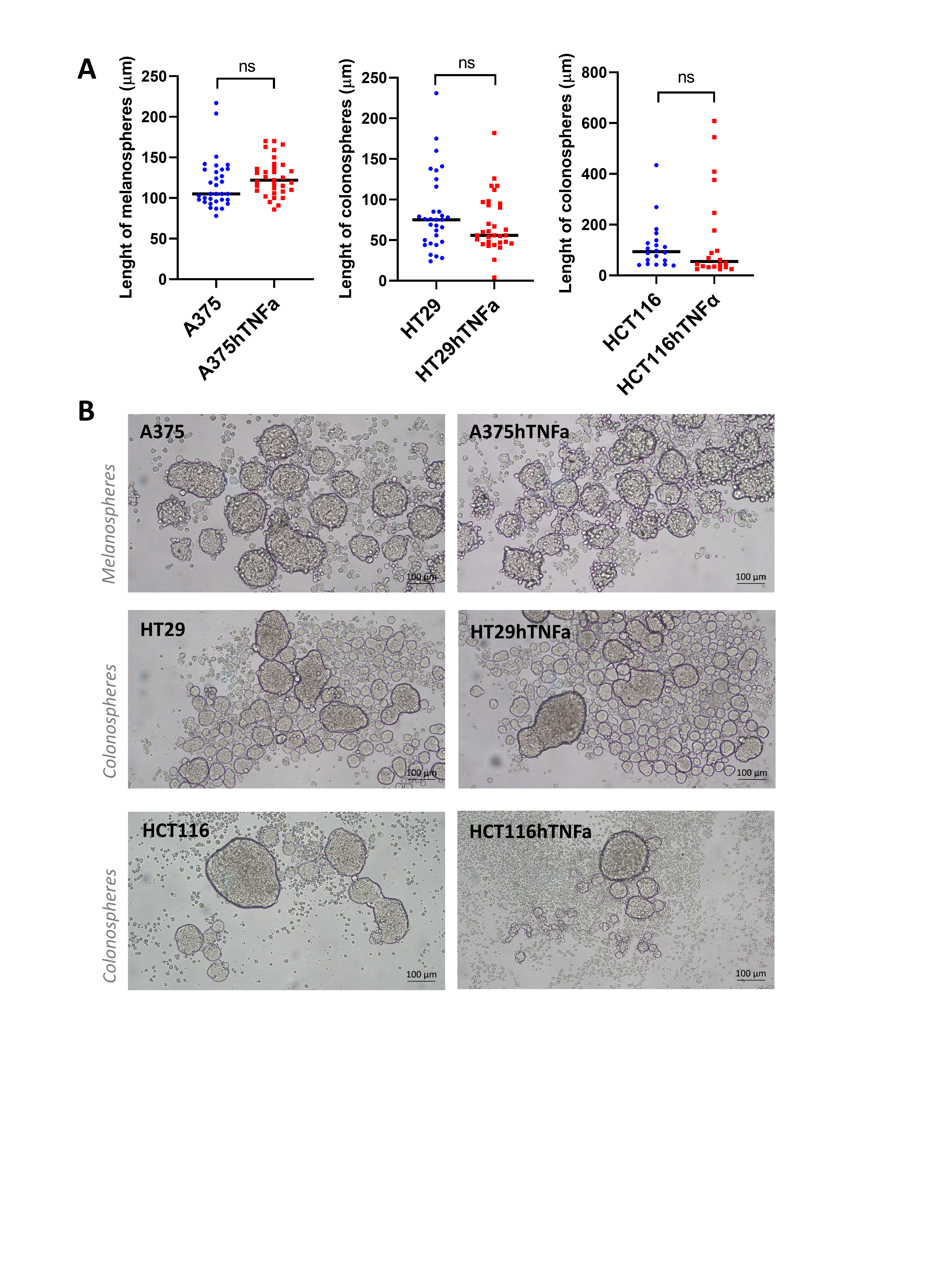

Supplement: Supplementary file 4 — Additional file 4: Figure S4. Spheroid formation ability is not affected under the TNFα overexpression. Spheroids of melanoma cells A375 (melanospheres) were grown in passage 0 for 4 days; spheroids of colorectal carcinoma cells HT29 and HCT116 (colonospheres) were grown in passage 1 for 3 days. Colonospheres later quickly fused together into big spheres with length of more than 600 μm. (a) Lengths of spheres; bars represent medians, n = 20–30, Mann-Whitney test; (b) representative light microscope images, scale bar: 100 μm. [file 12885_2021_8237_MOESM4_ESM.tif]

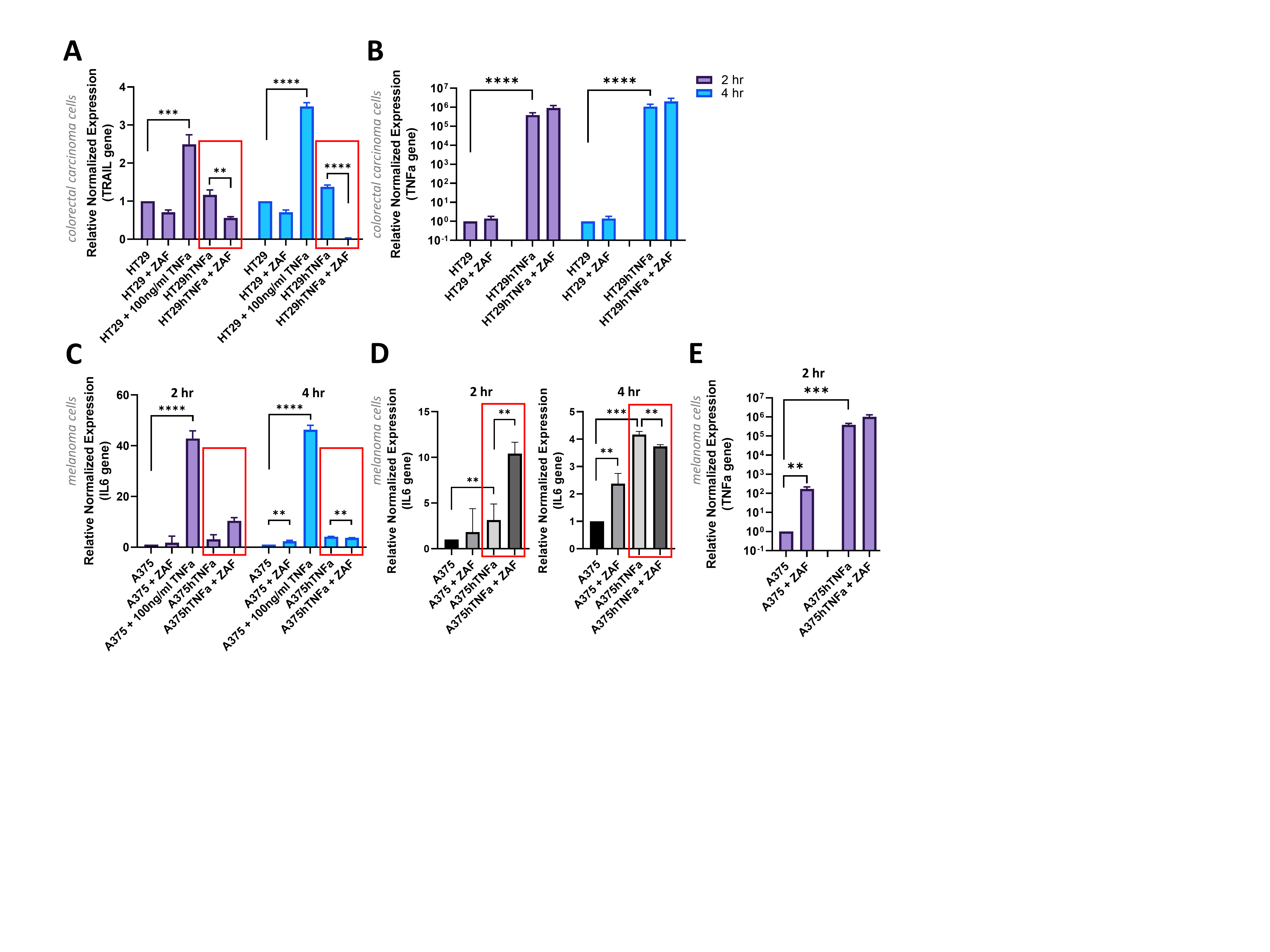

Supplement: Supplementary file 5 — Additional file 5: Figure S5. Inhibitor of TNFR1 receptor zafirlukast (ZAF) inhibits (a) TRAIL gene overexpression in engineered colorectal carcinoma cells HT29hTNFa and (d) IL6 gene expression in malignant melanoma cells A375hTNFa (highlighted with red line). (e) Zafirlukast itself also significantly increases TNFα gene expression in parental cells A375, so its inhibitory effect on IL6 overexpression (d) is evident firstly after 4 h post-treatment. (a, c, d) Overexpression of TRAIL and IL6 confirmed in TNFα overexpressing cells and in control cells treated with recombinant TNFα (100 ng/ml). Cells were treated with zafirlukast (100 μM) for 1 h at 37 °C; recombinant TNFα (100 ng/ml) was then added to the control samples for 2 and 4 h (2 h, 4 h) post- zafirlukast treatment and then all cells were immediately harvested for reverse quantitative PCR for TRAIL, IL6 and TNFα gene. Expression normalized to HPRT1 reference gene; values are mean of triplicates ± SDs; unpaired t-test was used to statistical analysis. Pictures (c) and (d) represent independent experiments. [file 12885_2021_8237_MOESM5_ESM.tif]
